# Why we must face our past: reconciliatory solidarity for global health ethics

**DOI:** 10.1136/bmjgh-2025-022373

**Published:** 2026-03-18

**Authors:** Ming-Jui Yeh, Po-Han Lee

**Affiliations:** 1 Institute of Health Policy and Management, National Taiwan University, Taipei, Taiwan; 2 Department of Public Health, National Taiwan University, Taipei, Taiwan; 3 Global Health Program, National Taiwan University, Taipei, Taiwan

**Keywords:** Global Health, Interdisciplinary Research, Health policies and all other topics, Violence, Gender-Based Violence

## Abstract

This article proposes a cosmopolitan theory of global health ethics based on reconciliatory solidarity at both local and global levels. The proposed theory provides the ethical and empirical grounds for the moral imperative of global health solidarity that is often called on today. Reconciliatory solidarity requires that a people/nation-state address the historical injustice and the legacies of political violence within its boundary, with the social connection model suggested by the political philosopher Iris M Young. Reconciliatory solidarity has advantages over the prevalent human rights-based approach and utilitarianism in addressing historical injustice. Through the rectifying efforts, true parochial reconciliation would be possible at the local level, serving as the prerequisite for reconciliation beyond national borders. With a fair number of well-ordered societies and nation-states, cosmopolitan reconciliation and genuine global solidarity would be possible.

**SUMMARY BOX**

- Global health advocacy and actions are often based on the call for global solidarity, which has long lacked the ethical and empirical grounds.
- The paper offers a more balanced and plausible alternative to the present dominant discourses of human rights and countries’ self-interest.
- The proposed approach of reconciliatory solidarity between parochial and cosmopolitan perspectives would provide more substantial ethical justifications for global health actions.
- Amidst the rising populist and polarised political landscape and ever-surge of suspicion on science, the article proposes a timely and viable alternative for public health academics and practitioners around the globe.

## Introduction

 Global health’s calls for ‘solidarity’ will remain fragile unless grounded in domestic, forward-looking reconciliation that openly addresses historical injustice; once such ‘reconciliatory solidarity’ is institutionalised within states, it can scale outward to durable solidarity beyond borders that better sustains health cooperation and redistribution. The ideals of global health ethics are grounded in an empirical basis of international solidarity, which implies that peoples around the globe are willing to bear the necessary costs to uphold a world in which we are interconnected and depend on each other, taking actions of mutual assistance and the redistribution of resources at the global scale.^1 2^

Solidarity is frequently invoked in global health. Some appeal to countries’ self-interest in collaborating to address global health threats.^2 3^ Others appeal to universal rights or common morality among humans or the ethical global society.^4^ What is underspecified is its source of legitimacy and political feasibility.^5^ Jennifer J Prah suggests that there could be public moral norms in pursuing health equity. If such norms could be internalised by peoples, a more just global health arrangement would be possible; nonetheless, Prah distinguishes her provincial globalism model of global health justice from the concept of solidarity for its ‘us-versus-them’ mentality and the lack of clear mandates on obligations for justice.^6^ The article concurs with Prah’s diagnosis on the limit of solidarity, but maintains that it is still ethically relevant and politically feasible to consider the role of solidarity in justifying global health actions.

This article aims to develop a theory of global health ethics grounded in a *cosmopolitan* reconciliatory solidarity, which is constituted by the *parochial* (in contrast to the cosmopolitan) reconciliatory solidarity in each society. Parochial reconciliatory solidarity is defined as a forward-looking social commitment rooted in public acknowledgement, accountability and structural reform to address historical injustice. The solidarity is ‘parochial’ in that it is limited to the boundary of a single polity and the society it consists of. It binds a polity’s diverse members into a workable ‘we’, enabling outward-facing solidarity, which, as argued later, means institutionalised, justice-oriented extension of solidarity beyond a state’s borders, manifested as predictable resource-sharing and risk-sharing co-governance with non-citizens affected by its actions. It differs from charity (discretionary) and bare cooperation (transactional) by treating cross-border commitments as duties.^7 8^

Such outward-facing solidarity is most durable when grounded in prior reconciliation-based civic solidarity at home, which lowers identity conflict costs and builds political willingness to support equitable global health commitments. With an adequate number of polities reaching parochial reconciliation, a cosmopolitan reconciliatory solidarity among them would be possible. The mechanism is twofold. First, instead of a direct, cosmopolitan sentiment or values, territorial or national solidarity should serve as the parochial grounds for the broader scope of solidarity, ie, global solidarity. Taking parochial grounds for global solidarity is ethically preferable and empirically more plausible. The key point is to overcome the xenophobic nature of parochial solidarity within and of the polity.

Second, therefore, to forge a sense of solidarity that extends beyond territory or national borders, it is necessary to first look back to a country/nation-state’s own history and reconcile with the unjust part of it. To really look into the history, honestly acknowledge historical wrongs and respect the oppressed groups affected by them could enable people (the oppressors, the oppressed, the ‘indifferent’ others and all their descendants in future generations) to envision a more universal humanity and shared values. This is where genuine reconciliation between conflicting memories within a society’s subpopulations is possible. When the *parochial* reconciliation solidarities have been reached in historically interconnected states and peoples, the *cosmopolitan* reconciliation between states and peoples at the global level would be made possible, and ultimately, a genuine global solidarity would be possible.

**Figure 1:**
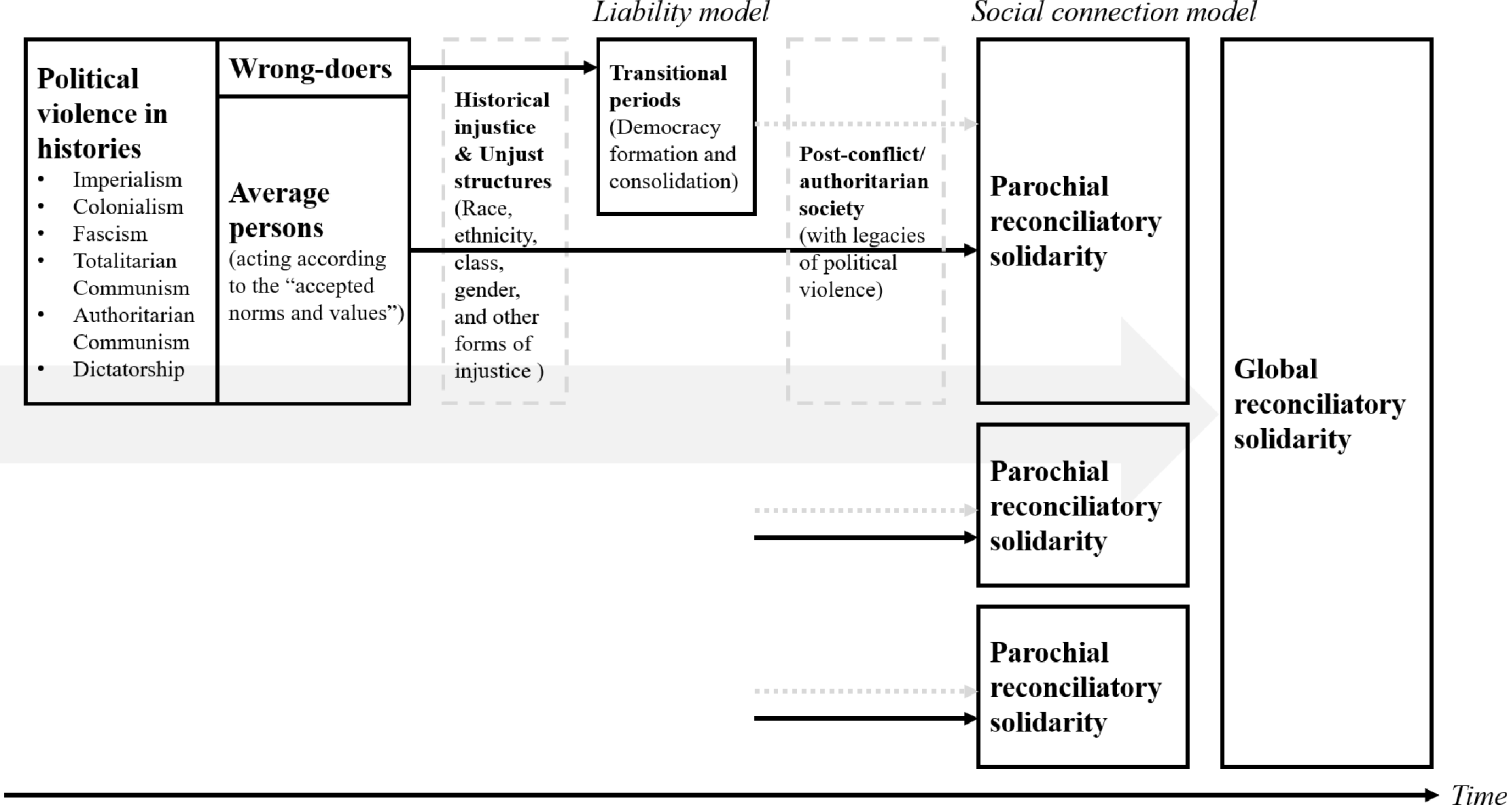
Parochial and global reconciliatory solidarity. Note: The solid and dotted arrows denote the explicit and implicit effects of political violence derived from historical injustice at the local level over time (parochial reconciliatory solidarity). The grey wide arrow denotes the impact of political violence at the global level (global reconciliatory solidarity). The details in the second and third local cases are abbreviated for simplicity. MJY draws the figure.

The article starts by discussing historical injustice within a modern nation-state, calling it local historical injustice and analysing its major sources, the consequent political violence and health harms. In the next section, the article argues that the social connection model proposed by Young^9^ would be a suitable conception of responsibility for rectifying historical injustice and proposes a possible mechanism by which the model would make democratisation and transitional justice practice more politically feasible by forging parochial reconciliatory solidarity. South Korea’s experience is used as an illustrative case to demonstrate the mechanism’s feasibility. Then, it is argued that how local historical injustice is dealt with (rectified through the reconciliatory solidarity) is related to global historical injustice and why global solidarity depends on it. The conceptual framework of the analysis of local and global historical injustice and reconciliatory solidarity is shown in figure 1. The article then discusses why reconciliatory solidarity is a better approach to realising cosmopolitan ideals and forging global solidarity than the human rights-based approach and other cosmopolitan theories of global ethics. The article concludes with policy implications for health reformers and advocates.

Two caveats must be put up front. First, reconciliatory solidarity alone does not specify the details of actual transitional solidarity practice or the principles of redistribution at the policy level. There are already abundant scholars devoted to this issue. The concept aims to provide a theoretical and structural underpinning to guide policies, depending on the specific context in each state and in each case of global health collaboration. Second, the analysis in this article primarily applies to, following John Rawls’s classification, well-ordered societies that are (largely free and democratic and, in some cases, hierarchical but still well ordered)^10^ and in peaceful relationships today. While the aim is to justify global health solidarity for global health actions, the scope of collaboration with other regimes has ethical limits. Well-ordered societies should not, and ethically cannot, collaborate with dictatorships or totalitarian governments that are clearly responsible for aggression, genocide or crimes against humanity. However, under the social connection model, the absence of direct collaboration does not exhaust responsibility. States that benefit from unjust global arrangements, or that remain silent in the face of structural harm, are not exempt from responsibility; rather, such silence constitutes a form of participation that grounds forward-looking obligations proportional to their power, privilege and capacity to act, including through non-coercive, multilateral and institutional forms of global health cooperation.

Note that the analysis has an assumption about the boundary of the existing society. In reality, there is a problem of uncertain or dynamic boundaries between societies, such as disputed territories and the movement of large numbers of people (emigration/immigration/refugees) for various reasons and across different periods. While the analysis here does not assume a homogenous population within each society (as the internal tensions due to heterogeneity are precisely what the proposed theory aims to address), it has to, for the moment, assume relatively stable boundaries as the baseline of discussion. Immigration, for example, under today’s international order, is subject to the discretion of the absolute power of sovereign states. The article does not dispute this. However, if immigration leads to diverse subpopulations that cause severe internal tension within society, then it would be in the interest of the parochial reconciliatory theory.

It is important to clarify that the argument advanced here is not a gatekeeping claim about which societies deserve global solidarity. Instead, it is a sequencing argument about political feasibility and institutional durability. The claim is not that societies must complete domestic reconciliation to qualify for global solidarity, but that reconciliation-grounded civic solidarity tends to generate social trust, legitimacy and willingness to bear costs that make sustained global health obligations politically possible. In many cases, domestic reconciliation itself presupposes transnational responsibility, particularly when historical injustice was produced by colonialism, imperialism or the global political economy. Reconciliatory solidarity, therefore, describes a capacity-building pathway rather than a moral precondition for durable global cooperation.

## Historical injustice and political violence

Historical injustice is a type of injustice that originates from the past. While how long is enough for the past to be considered a part of the ‘history’ is a subject of debate, for the sake of the analysis, the article adopts a view that refers to the past as the political stages previous to a state’s current form of government within the boundary of a single society, called local historical injustice. The wrongs committed by governments and organised political entities in the past have caused long-lasting harm that persists to this day. This specific form of historical injustice is political violence. According to the WHO’s definition, political violence is a form of collective violence, including “war and related violent conflicts, state violence and similar acts carried out by larger groups” with motives such as “committed to advance a particular social agenda includes, for example, crimes of hate committed by organized groups, terrorist acts and mob violence” (p. 6).^11^ In recent human history, imperialism, fascism, totalitarian and authoritarian communism, colonialism, dictatorship, among others, are familiar sources of ideologies that have driven governments to literally murder, torture, imprison and deprive people of freedom, property and lives.

Compounding with other social and cultural historical injustice, such as race, ethnicity, social class, gender factors and political violence in the past would continue to cause harms to the people through (1) its direct effects (physical and mental harms and disabilities) on the people, (2) maintaining an oppressive social structure and (3) reproducing conflicting identities and historical memories, which may lead to political violence in the future. First, the direct effects are well evidenced. Besides its direct life-causing effects and socioeconomic impacts, political violence has been an important determinant of physical and mental health, disabilities and deaths.^12^,^18^

Second, the oppressive social structure created by political violence, such as poverty, social exclusion, stigmatisation and marginalisation,^19^ would further hinder the formation of a society from forming common values and a shared solidaristic sentiment. Even if the state does not perform political violence today, the oppressive social structure may be maintained, continuing to reproduce the unequal opportunity for self-development and self-determination between the subpopulations that have different historical roles in the past injustice.

Third, the reproduction of different identities and historical narratives is an even more complex and contested process. During periods of political transformation—such as civil wars, democratisation, decolonisation, liberation or post-conflict reconstruction—social groups may experience these processes unevenly, giving rise to differentiated perceptions of loss, responsibility and entitlement. These dynamics do not necessarily produce coherent or unified ‘winner’ and ‘loser’ groups; rather, they often generate fragmented, competing narratives within and across subpopulations. Over time, such narratives can become embedded in social memory, political discourse and institutional practices, shaping how groups interpret both past injustice and present social positions.^20^ When left unaddressed, these contested narratives may acquire heightened political salience and contribute to enduring mistrust or antagonism between groups, complicating efforts to build inclusive forms of civic or parochial solidarity.

If reconciliation processes are sufficiently effective, or if political institutions succeed in stabilising relations among groups over time, historically differentiated identities may cease to function as primary sources of social conflict, even though they do not disappear. Identities and collective memories are not simply erased by the passage of time, but are continuously reproduced through institutions, education and power relations. What changes is not their existence, but their political salience. Where reconciliation has been partial, contested or absent, coexisting identities are more likely to harden into antagonistic positions informed by competing historical narratives and asymmetric power relations, making parochial solidarity difficult to sustain. Under such conditions, historical memory becomes a site of political mobilisation, and conflicting narratives, when activated through political manipulation or crisis, can contribute to renewed political violence. The persistence of civil wars, genocides and large-scale social and political exclusion in the 21st century illustrates how unresolved historical injustice, rather than mere diversity of identities, continues to pose risks to social cohesion. Reconciliation, in this sense, should be understood not as the elimination of difference, but as the institutional containment of conflict under conditions of political equality.

These three mechanisms of harm caused by political violence extend the effects and legacies of historical injustice, consolidate unjust social conditions and continue harming people in today’s peaceful and democratic society. Under today’s humanitarian and human rights standards, at least in democratic societies, people would no longer accept the violent, bloody dispute-resolution processes that were prevalent in history. Therefore, the challenges for today are to determine how people should act to address the persisting harm caused by historical injustice and what corresponding political actions they should take to prevent political violence and collaborate on global health and peace. The job for normative theories is to take the first step by identifying who should be responsible for justice.

## Allocating responsibility for justice and forging parochial solidarity

While Young’s social connection model has been subject to debate, particularly regarding the scope and demandingness of forward-looking responsibility, it remains one of the most influential accounts for addressing structural and historical injustice without collapsing responsibility into individual blame. As suggested by Young, the responsibility for justice, including the rectification of historical injustice, could be determined by two different conceptions of responsibility, namely, the liability model and the social connection model.^9^

The liability model holds that wrongdoers should be held responsible for the harm they cause. Victims of wrongdoing have the right to claim compensation or retribution. Under this model, the key to determining responsibility lies in identifying the wrongdoing actions and the harmful outcomes, and in establishing the causal relationship between the two. The liability model is the basic approach that holds the historical wrongdoers accountable. The wrongdoers are identifiable because their actions directly caused harmful outcomes. For instance, if a dictator ordered the massive killing of political prisoners, the liability model would identify and prosecute the wrongdoers and return or compensate the property that the victims had been deprived of. These activities are standard transitional justice practice. Nevertheless, in the past, many collaborators and ordinary folk were implicitly or passively collaborating with the wrongdoing government for their own benefit, out of fear or out of political alienation.

Determining the causal relationship between the harmful outcomes and wrongdoers’ actions or inactions (actively failing to do something that should be done for justice), or even non-actions (passively ignoring or disregarding what was happening),^21 22^ is much more difficult. Yet, it is also challenging (if not impossible) to rule out their contribution to the harmful consequences. In addition, while the wrongdoing government has been terminated and people may today live under a normal government, the legacies of past political violence may persist, and the oppressive social structure is still reproduced, within which, nevertheless, no specific wrongdoers can be identified. If the net of liability is too expansive, it may exacerbate conflicting identities and historical memories, potentially inducing political violence in the future. In situations like these, where average and ordinary persons only acted according to the “accepted rules and norms” (p. 52)^9^ at that given time, the liability model meets its limitation. It could hold no one accountable, yet there are clearly blunt, unjust harms. Young suggests adopting the social connection model instead.

The social connection model identifies the responsibility in a very different way. It does not seek to establish a causal relationship between individual wrongdoers and the harms resulting from their wrongful actions. It focuses on the background conditions that create harmful and unjust outcomes. In such situations, most people acted according to the ‘accepted rules and norms’, yet their actions were interdependent with each other’s, all part of what constitutes the social structure. Under the social connection model, responsibility derives from the social structure’s participation in causing harmful outcomes. Therefore, no individual may be found responsible for the wrongful actions (in the sense of the liability model), but every individual participating in the structure would be responsible for the harmful outcomes it causes. Under the social connection model, the collective basis of responsibility is people’s interdependence and collective participation in the social structure that causes detrimental outcomes.

By de-linking causal inference at the individual level and refocusing it at the structural level, the social connection model diffuses responsibility to a broader population that would otherwise be innocent under the liability model. This approach is suitable for addressing historical injustice, where no apparent wrongdoers (or those who committed past wrongs have long died, and their descendants do not commit wrongful actions) are identifiable. Yet, there are apparent injustices and continuing harm today.

In the social connection model, responsibility for justice is not determined by identifying causes and guilty wrongdoers, but by the four parameters of power, privilege, interest and collective ability, as evaluated in the current time and space. While these parameters may align with the distribution of subpopulations and thus hold the allegedly wrongdoing-related subpopulations in history more accountable, this is not necessarily the case. Even if ethically, they should bear more responsibility, it is not because they are accused of wrongdoing or wrongdoing-related and hence held accountable, but because they still occupy a more advantageous position in society today due to historical legacies. With more power, privileges and retained advantageous positions, they should contribute more to rectifying historical injustice.

For instance, if an ethnic group benefited greatly during the colonial period, forming a solid business and trading network that persists to this day, they may occupy advantageous social positions, having greater power to influence policies and greater privilege to enjoy the benefits of economic growth, compared with other ethnic groups. Then, this advantageous ethnic group should have a larger share of responsibility today. In other words, the social connection model stops seeking who is guilty and treats all subpopulations equally in this regard: no one is guilty, and everyone is responsible for participating in the present oppressive social structure.

With this conception of responsibility, differences and the confronting historical interpretations between subpopulations would be mitigated, and transitional justice practices and institutional reforms aimed at preventing political violence from recurring would be more palatable to people across all subpopulations. The historically alleged wrongdoing-related subpopulations do not have to fear being held individually accountable, found guilty and prosecuted by the transitional justice regime, which they may otherwise consider a form of political violence imposed by the current ‘liberal and democratic’ government. Historically suffering, victimised subpopulations may find this model less just through the lens of strict retributive justice. However, they could still benefit from living with less resentful neighbours. This benefit is particularly salient for situations with no exact, distinct geographical boundary between the allegedly wrongdoers and the survivors.

For example, if supporters of and those oppressed by the past authoritarian government were not distinguishable by ethnicity or geographic distribution, the potential internal tension would be pervasive. People on both sides would not have their own clear, safe zones if attempts at retribution, guided by the liability model, provoked internal tensions. They are neighbours who have to live together, go to the same grocery stores, send their children to the same schools and work in the same places. Under the social connection model, a reconciliatory and peaceful sphere would be created among the entire neighbourhood. There would be no need for safe zones on either side.

Again, the social connection model applies only to those who acted in accordance with the ‘accepted rules and norms’ at the given historical periods. It would not let those actual wrongdoers, who are to be found guilty, easily get away with their own responsibility under the liability model. Obvious cases like executions, tortures, rapes and civilian killings in colonies, wars, armed conflicts or terrorist attacks (with clear political purposes)^23 24^ and gender-based violence (as a part of political violence)^25^ definitely have guilty wrongdoers to be held accountable. They should be subject to lustration, prosecution, deprivation of unjustly procured property or other penalties as an integral part of transitional justice practice. The liability model and the social connection model are complementary and are not substitutes for each other in precise and actual cases of wrongdoing actions and guilty individuals.

Indeed, there is still a question regarding how a society should identify who should be counted as ‘actual’ wrongdoers. Collaborations and behaviours that take advantage of the wrongdoing system are hard to distinguish from merely acting in accordance with the ‘accepted rules and norms’. Should those civil servants who followed their orders be considered guilty or acceptable? The controversial case of the Nazi German officer Otto Adolf Eichmann, as reported by Hannah Arendt, is a good example.^26^ Eichmann was a high-ranking officer; what about the other lower-ranking civil servants or privates? International criminal tribunals may have addressed such concerns in particularly extreme situations, such as the case law developed by the International Criminal Tribunal for the Former Yugoslavia (ICTY), the International Criminal Tribunal for Rwanda (ICTR) and the International Criminal Court (ICC) regarding the actual control of the matter at issue and its effect^27^; the question remains unresolved in a universal way across jurisdictions in less extreme circumstances.

In addition, outside of the state apparatus, should merchants making huge profits (usually related to some political privileges and bribes for officials) from the wrongdoing system, eg, an opportunistic-only version of Oskar Schindler who profited from the work of the political prisoners, be considered guilty? These scenarios fall around the borderline of guilt and are normally acceptable. Young did not leave enough hints in her unfinished book to answer this question when she died in 2006 (the book^9^ is an edited posthumous manuscript). Nevertheless, given the forward-looking spirit of the social connection model, one could reasonably infer that, in principle, the scope of identifying wrongdoing should be narrow and adhere to a strict causal relationship between actions and harmful outcomes.

The social connection model brings about a forward-looking conception of responsibility that, despite arguably being conservative and seemingly compromising with political reality, forges a homogeneous unity and a just-seeking, solidaristic sentiment among the entire population—a parochial, reconciliatory solidarity. The transitional justice practice that prevents future political violence would be more feasible in this political atmosphere. The democratisation and democracy consolidation processes (after the state has undergone democratisation) would also be more politically viable because subpopulations, once divided by their relationships with historically wrongdoers and the corresponding identities and historical memories, would now be bound together in pursuit of a just society. They may still challenge each other’s identity or memory, but they will not dispute each other’s recognition of historical wrongs. They will also be less likely to attempt to exacerbate populist mobilisation and commit political violence against each other if they control the state apparatus through a legitimate, fair democratic process rather than cause democratic backsliding.^28^

In sum, with proper transitional justice practice right after or alongside the democratisation process and the consolidation of democracy in later years under the social connection model, a true reconciliation would be possible between the once-conflicting identities and historical memories among subpopulations of society. This parochial reconciliatory solidarity would be integral to territorial or national solidarity within the national border. It would enable individuals across all societal subpopulations to envision themselves as equal and free social members, or fellow citizens. With this sense of equality^29^ and shared identity^30^ in mind, people may tend to recognise each other’s health and social needs when institutional reforms, public acknowledgement of past injustice and credible accountability mechanisms are in place.^31^

Therefore, parochial reconciliatory solidarity would overcome the existing conflicts between national identities and ethnic differences, underpinning mutual assistance, redistribution and collective action required to fulfil the health and social needs of all people in society, thereby strengthening the welfare establishment in the face of population ageing and economic stagnation. The completed traditional justice practice, the consolidated democracy and the strengthened welfare system would ultimately contribute to better health outcomes for the population in society.

### The case of transitional justice in South Korea

The case of South Korea, formally the Republic of Korea, may be an illustrative example that is close to the ideal of parochial reconciliatory solidarity. South Korea underwent democratisation in the late 1980s and enacted a series of transitional justice laws during the 1990s and 2000s.^32^ These transitional justice practices address the historical wrongs in the previous South Korean authoritarian government as well as those in the earlier Japanese Empire Occupation before World War II (WWII), containing a decolonising purpose.^33^ (The North Korea–South Korea relations, however, are a related but different political issue.)

During the same period, South Korea’s welfare and health systems were expanded, driven by the political competition^34^ and active civic movement^35^ under democracy. These political actions could be deemed social collaboration among free and equal citizens. The resulting expanded welfare arrangements represent the people’s willingness to care more about their fellow dwellers’ health and social needs. Other elements, such as the Asian economic crisis, Korean state capacity and the political will of politicians, of course, also contributed to the expansion, but the fact that South Koreans were less divided by the authoritarian politics—a status of parochial reconciliation—certainly plays an important role in forging the sense of equality and shared identity.

In South Korea’s case, people’s overall support for expanding public health and social care could be interpreted as a form of sharing responsibility for justice, seeking not to identify individual wrongdoing but to heal society and remedy unjust health outcomes in the present. Notably, amid intense public demand, South Korea is among the very few countries that have prosecuted and punished individual wrongdoers (though only at the highest levels of political leadership; unlike lustrations throughout the entire administration).^36^ Even the political successors of the former dictator cannot avoid admitting the historical wrongs in suppressing the democratic movement and initiating the corresponding truth investigation and compensation.^36^ Both the liability and social connection model could be observed in the transitional justice practice in South Korea, rightly demonstrating that the two models are complementary, not substitutive to each other.

The case of South Korea is particularly salient for the mechanism proposed here because most welfare states in the West were established and expanded in an earlier stage, right after WWII, during which the development was primarily driven by the national sentiments strengthened during the war and the economic and social arrangements (eg, the European Recovery Program) in the reconstruction era. Other East Asian countries with well-established welfare systems, such as Japan and Taiwan, might also be plausible cases. Nonetheless, Japan has much less internal tension, as it has completed the nation-building process long before WWII. Transitional justice is hardly an issue in Japan. Taiwan is in a far more complicated situation. While the government has initiated some transitional justice efforts,^37^ Taiwan suffers from multiple colonial histories and divided identities, let alone the external threat of aggression that further exacerbates the internal tension. Transitional justice is at best halfway in Taiwan.

Despite its transitional justice practices being subject to criticism, South Korea’s case suggests that properly addressed historical injustice would forge reconciliatory solidarity and potentially lead to collective actions to rectify unjust health and welfare conditions.

The mechanism described in this section is sufficient to sustain a single welfare state; however, the aim of this article also includes the global.

## Sustaining global solidarity with parochial ones

The parochial reconciliatory solidarity in each state/society is the ground of genuine global solidarity. At the local state level, the parochial reconciliatory solidarity sustains its welfare establishment and overcomes the xenophobic or exclusionary nature of typical national solidarity. The reasons are twofold: the presence of a common morality and the necessity of reconciliation beyond borders.

First, when reconciling with one’s fellow citizens under the social connection model, one would recognise the value of pursuing justice and rectifying historical injustice, forming a common morality. While the forms of historical injustice varied across societies, the goals are common: people are pursuing liberty from the state’s political violence and from oppressed social structures. The notion of common morality employed here is intentionally thin and procedural rather than value-thick or culturally comprehensive. It does not presume deep ethical consensus or shared traditions; it refers instead to a minimal recognition of equal moral standing and the unacceptability of systematic political violence—an overlapping normative baseline that enables coordination across difference rather than moral uniformity. No reasonable person who has lived in a free and democratic state today would be willing to live in such a situation ever again. This common goal suggests a shared morality or universal humanity among most humans, regardless of the form of historical injustice in each society. The common morality referred to here is used in a descriptive sense, presenting the set of morality, or to use Michael Ignatieff’s term, ordinary virtues,^38^ that underpins the human society as a whole, despite the enormous differences between each human society. The people would therefore further recognise that this common morality is not only limited to themselves (fellow citizens, conationals, ‘we’), but should also extend to ‘others’. There are two types of perceptions of ‘others’, namely, the others within and the others outside. The others within refer to non-citizen fellow dwellers living within the national borders. The others outside refer to those living in other states. The health and social needs of these others are at least worth considering to the degree that they are concerned about the presence of common morality.

Note that the significant challenges to cultivating common morality differ for the two types of ‘others’. For the others within, xenophobic and exclusionary sentiments constitute the primary obstacle. While local economic production may already rely heavily on the labour of these non-citizen residents, cultural, religious and racialised differences often prevent their recognition as moral equals with comparable health and social needs. Even in societies that have achieved a relatively high degree of parochial reconciliation, such exclusion persists. South Korea, for example, despite its transitional justice efforts and welfare expansion, continues to exhibit xenophobic sentiments^39^ and institutional discrimination against migrants, including in the provision of essential public health services during the COVID-19 pandemic.^40^ Intriguingly, the Korean identity seems to be a preventive rather than an enforcing factor for the xenophobia towards immigration,^41^ suggesting that the somewhat homogenous imagination of the people might lead to an inclusive attitude.

The persistence of such exclusion does not undermine the relevance of reconciliatory solidarity; rather, it illustrates the limits of any solidarity framework and the need for internal corrective mechanisms. Unlike nationalist solidarity, which tends to naturalise insider–outsider distinctions, reconciliatory solidarity contains a reflexive dimension: by confronting historical injustice and structural privilege, it exposes how exclusionary boundaries are politically produced rather than morally given. This reflexivity does not eliminate xenophobia, but it renders it contestable within a shared civic framework.

For the others outside, the primary obstacles are psychological, emotional and geographical distance, limited perceived relevance and historically structured international relations, including colonial legacies. In these contexts, reconciliatory solidarity facilitates outward extension by linking domestic experiences of injustice, responsibility and reform to analogous conditions beyond national borders. South Korea again provides an illustrative example. Following democratisation and the expansion of domestic health and social care systems, South Korea gradually transformed from an aid recipient into a contributor in global health and humanitarian governance.^42^ While such engagements inevitably involve geopolitical considerations, they also require sustained public willingness to bear real costs, including fiscal redistribution and participation in United Nations peacekeeping operations.^43^ South Korea thrives not only to be a responsible member of the international community, but also in a mutual assistance sense by ‘giving back’ to the ‘others’ that had once helped them.^44^ These outward-facing commitments are more plausibly sustained where citizens have internalised responsibility for justice at home.

Despite persistent challenges in including both internal and external ‘others’, parochial reconciliatory solidarity offers a more plausible foundation for global health solidarity than human rights–based or abstract cosmopolitan approaches. By embedding moral recognition, responsibility and cost-bearing within a shared political community before extending them outward, reconciliatory solidarity provides institutional and motivational resources that those approaches lack.

Second, parochial reconciliatory solidarity is a precondition for reconciliation beyond national borders. In many situations, historical injustice is caused by factors or actors beyond existing national borders—what is called global historical injustice. For instance, former imperial colonisers who controlled colonies that have become multiple independent states, or former authoritarian/totalitarian states that were dissolved into various smaller states, were historical wrongdoers; many individuals were involved in collaboration or complicity with them. Liability or the social connection model within a state’s jurisdiction cannot address these actors, and it requires reconciliation across existing national borders. So far, very few transitional justice practices have been carried out beyond national borders that could hold wrongdoing states accountable. At most, international criminal tribunals and courts may hold individual wrongdoers accountable. Some argued for the international criminal justice system to be treated as a globalised form of transitional justice, a transnational extension of accountability that complements truth-seeking, reparations and institutional reform rather than substituting for them.^45 46^ Transitional justice practice at a global level is indeed extremely difficult. Nevertheless, for reconciliation across borders to be possible, reconciliation within the borders is a prerequisite. The local people must first recognise the common morality against the historical injustice in their local context to identify potential wrongdoing or the accepted rules and norms at the global level.

Reconciliation-grounded civic solidarity within a polity establishes a workable common morality and creates the conditions for cross-border reconciliation. As more polities build this at home, they become able and willing to extend care outward as an obligation, sustaining mutual assistance, redistribution and collective action globally. This yields a cosmopolitan form of global solidarity with both normative and empirical foundations and is therefore more durable. Global health action grounded in this kind of inside-outward-facing solidarity is ethically stronger and politically sustainable: it treats cross-border cooperation as duties of justice rooted in domestic reconciliation, which enhances willingness to contribute.

While not (yet) peremptory, strands of customary international law already point this way. The International Court of Justice has recognised obligations *erga omnes* owed to the international community (*Barcelona Traction*, 1970) and later affirmed the peremptory status of core prohibitions (*Belgium v. Senegal*, 2012). It has also identified good-faith duties to negotiate (*Legality of Nuclear Weapons*, 1996) and procedural duties of cooperation and environmental assessment to prevent transboundary harm (*Gabčíkovo–Nagymaros*, 1997; *Pulp Mills*, 2010). The case of South Korea’s gradual participation and support for global collaborations discussed above is also a good example. It shows that a sense of duty to respond and to assist in global health and broader global humanitarian challenges could be mutually cultivated at the national level.^44^ This willingness is possible with the condition that South Koreans have reached a (relatively) parochial reconciliatory solidarity.

The article extends these logics to health by specifying positive duties of protection, fair terms and co-governance in public and global health. These are the domains where treaty and custom remain thin, due to the structural disconnect between domestic and international orders that features modern international law.^47 48^ Our proposal for global health ethics, thus, traverses the two planes long mistaken to be independent and separate and grounds the pursuit of justice in social connections among actors, reconciliatorily from the inside out, that go beyond the outside-in, top-down or a purely deontological framing.

## Reconciliatory solidarity: a better alternative to cosmopolitan theories

A reasonable objection is that developing a theoretical account of global health ethics is unnecessary because its aims appear already covered by the long-standing, well-established human rights-based approach and related cosmopolitan frameworks such as universal health coverage and the Sustainable Development Goals. For simplicity, the analysis here focuses on the human rights-based approach and cosmopolitan utilitarianism, as the underlying logic is similar.

The human rights-based approach has maintained a common morality at the global level. However, given its highly centralised nature, the extent to which peoples across different states have widely accepted human rights is a major question. In many places, it could be argued that the human rights-based approach is essentially an elite initiative and lacks social consensus or public political support. In addition, many states do not sign all the human rights treaties or covenants. In these states, the legal nature of the human rights-based approach limits its applicability. Even for the state parties of these treaties or covenants, the bearer of the ethical (and legal) obligations would be the state governments. The state may resolve the present human rights violations, fulfil the realisation of health and social protection and even prevent political violence in the future; it may fail to address the historical wrongs and injustice due to the legal principle of non-retroactivity and, therefore, no responsibility arising from the wrongs committed when an obligation had not existed yet. In other words, the human rights-based approach is only forward-looking and can provide minimal, if any, implications for the past. Therefore, a society divided by conflicting historical memories will continue to suffer from past injustices and their enduring effects. These two reasons, the top-down centralised and legal natures, make it politically less feasible. The elite-initiated, top-down nature renders the human rights-based approach ethically less preferable, for it lacks democratic (Young would argue)^9^ and solidaristic (Prainsack and Buyx would argue)^1^ grounds.

Peter Singer’s cosmopolitan version of utilitarianism is also a commonly referenced theory supporting global redistribution for global health.^49 50^ It argues for the maximisation of happiness (and health utility, in this particular discussion) through a pragmatic approach, suggesting that everyone in the more developed part of the world should donate to saving lives to the extent that they can afford. This argument could reasonably be applied at the international level, where each state has an ethical obligation to help less developed counterparts to the extent it can afford. Singer implicitly relies on human compassion as the ground of this utilitarian ethical obligation. The problem with this approach, again, lies in the empirical aspect. Even if one does not dispute how Singer derives the ethical argument from empirical assumptions about human nature, one could still maintain that, in reality, for this kind of human nature to be truly enacted at the population level, some forms of solidaristic sentiment shared across different subpopulations and peoples are still required.

Hence, the debate returns to the article’s initial query: how could parochial solidarity overcome its parochial or xenophobic nature and become the grounds for global solidarity? The answer would be that, as argued, it would first require facing historical injustice and pursuing parochial reconciliation and solidarity, then extending it beyond national borders. While the human rights-based approach’s perspective, treaty-centred design under-addresses historical legacies, cosmopolitan utilitarianism depends on exposed altruism and often lacks institutionalised pathways for sustained contribution. Our account helps repair both by pairing memory work and trust-building with reconciliation-grounded social connection, both internally and externally.

## Conclusion

Drawing on the social connection model that addresses historical injustice and political violence, the article proposes a cosmopolitan theory of global health ethics based on reconciliatory solidarity at both local and global levels. With enough parties involved having reached parochial reconciliation within their own boundaries, cosmopolitan reconciliation and genuine solidarity beyond the national borders would be possible. The overarching imperative of global (health) solidarity would, then, be better justified than those appealing to the human rights-based approach or the utilitarian account, which ground their calculations in the entire human species.

The proposed theory has two major limitations. One is that the present arguments limit the focus on well-ordered societies and nation-states. Nonetheless, many of the causes of political violence and the historical injustice behind them today happen in those places with outlaw regimes, where dictators, totalitarian governments, aggressors and colonisers still dominate.^10^ How global solidarity could be forged between well-ordered and not well-ordered societies remains challenging for the reconciliatory solidarity theory and global health collaboration.

Second, the reconciliatory solidarity approach is methodologically nationalist. It deems the necessity of reconciliation among a group of local peoples or dwellers first before the possibility of reconciliation (and the rectification of historical injustice) between larger groups of people, such as between people living in developed, former empires and people living in formerly colonised societies. The article uses South Korea as an illustrative case to show that reconciliatory solidarity, both parochial and cosmopolitan, is possible but not without limitations. This case shows that xenophobic sentiment towards others within might be the most difficult to overcome. Each state or people has its own unique historical injustice to rectify, and the way they do so also lies in their traditions, cultures and social structures.

Some may rightly worry that this approach would place developing countries under a greater burden. While it is a fact that the current social and economic development stages may result from globalisation and colonialism, developing countries do not necessarily face more historical injustices or subpopulation tensions (and hence a greater burden) than developed countries. Again, the actual burden of pursuing reconciliatory solidarity would depend on the context. Therefore, the reconciliatory solidarity theory cannot provide more practical details about what an ethically more preferable or empirically more plausible mechanism might be. That is the question to be examined by those who dare to devote themselves to the greater health of human beings.

## Data Availability

There are no data in this work.
